# Exploring user experience of digital pen and tablet technology for learning chemistry: applying an activity theory lens

**DOI:** 10.1016/j.heliyon.2021.e06020

**Published:** 2021-01-22

**Authors:** Chwee Beng Lee, Jose Hanham, Kamali Kannangara, Jing Qi

**Affiliations:** a Western Sydney University, Australia; b RMIT University, Australia

**Keywords:** Mobile learning, Learning technologies, Activity theory

## Abstract

Mobile learning technologies are spreading rapidly in educational institutions throughout the world. Although research findings concerning the efficacy of mobile technologies for improving student outcomes are generally promising, there are still significant gaps in the research literature, particularly data from direct observational studies. This empirical investigation focused on how students made use of tablet devices and digital pens for learning Chemistry in an undergraduate university course. Observational data in the form of videos and static images, as well as, interview responses, were the main sources of data collected for the study. Activity theory was employed as the guiding theoretical framework to analyse and interpret the data. Several themes emerged from the data analyses, including the affordances of digital pen technology for facilitating reflective thinking, flexibility, peer collaboration, emerging learning and focused learning. It was also found that the use of these mobile technologies was contextualized, dependent on individual differences, and had challenges, for example, there was limited synchronicity between the operational design of the mobile devices and natural human movement. One of the main implications of the research is that when higher education institutions consider the potential benefits and challenges associated with mobile technologies they should take account of the interactions that occur between components within a system including, students, technological devices, and emerging learning processes.

## 1 Introduction

Mobile technologies are an integral component of teaching and learning in educational settings around the globe. For over a decade, there has been interest in unpacking relationships between use of mobile technologies and student motivation, learning and achievement outcomes (Attard and Holmes, 2020; Candeias et al., 2019; Helfin et al., 2017; Hsu and Ching, 2013; Mena-Guacas and Velandia, 2020). In the sciences, there is growing evidence suggesting that the effective deployment of mobile technologies can improve student learning outcomes (Sung et al., 2016). In neurobiology, the introduction of mobile devices in undergraduate courses has been linked to improved performance on neuroanatomy content questions (Morris2016). In physics, the use of mobile devices in high school settings has been associated with improved student learning (Zhai et al., 2019). In the natural sciences, mobile applications have been linked to enriching children's observations of the natural world (Kawas et al., 2019). Despite the accumulating evidence demonstrating the benefits of mobile technologies, it is important to acknowledge that if poorly implemented, mobile technologies are unlikely to result in improved student performance and may often be detrimental student performance (Zhai et al., 2019).

Mobile learning involves using technologies ranging from hardware such as smart phones to platforms such as virtual realities and a myriad of mobile applications (apps). For this study, we focused on digital pens coupled with mobile tablet computers, hereafter described as *table devices*. Based on an extensive literature search, we found very few studies focusing on the use digital pens for learning chemistry. Although some studies have reported some of the learning benefits of using digital pens, particularly for chemistry learning (Morsch and Lewis, 2015; Urban, 2017), we are yet to fully understand how digital pen technology influences chemistry learning. Consequently, our study intends to address this current research gap. We were interested in how learners take the initiative to learn with digital pens. Rather than imposing a set of prescribed collaborative activities, the teacher and students were encouraged to use the digital pen and tablet device in any way they felt appropriate. Our goal was to explore how learning emerges from the ground with the use of mobile technology.

Several researchers have recognized the value of activity theory as an analysis framework for mobile learning studies (e.g., Sung et al., 2016). Mobile learning research studies cover diverse technologies that contribute different affordances for users. Affordances refer to the “interactions between users and tool” (Wijekumar2006, p. 192), wherein the tool can enable or constrain users depending on their familiarity with the tool. In this study, the potential affordances associated with tablet devices used in conjunction with digital pens for learning were explored through the lens of activity theory.

The following research questions guided our inquiry:

⁃ Research question 1: In what ways does the tablet PC and digital pen technology facilitate students' learning of Chemistry?
⁃ Research question 2: What are students' experiences using digital pen technology in their learning?

## 2 Literature review

### 2.1 Digital pen technology and learning

There have been systematic reviews on the use of mobile devices in various educational settings (Crompton and Burke, 2018), ranging from K-12 educational settings (Crompton et al., 2017) to higher education and adult learning (Hwang and Tsai, 2011; Wu et al., 2012). Although these reviews have provided critical information on the use of mobile technologies in education, it appears that very few reviews have focused users' experiences with tablet devices together with digital pens. There is growing recognition that bodily actions can influence cognitive abilities (Macedonia, 2019). In theory, tablet devices, and particularly, digital pen technology have affordances that should encourage learners to utilise motor processes, for example, touching, dragging, tilting, moving, drawing and pointing (Wang et al., 2015). In science-based subjects, the physical manipulation of the environment is integral for allowing learners to connect abstract concepts to the observable environment. In the subject discipline of chemistry, concepts are abstract and complex, and developing students' ability to correctly draw structures, reactions, mechanisms and syntheses is vital to students’ comprehension of key concepts (Morsch and Lewis, 2015). As such, it can be argued that tablet devices and digital pens are important tools for promoting understanding of scientific phenomena. Interestingly, a small number of studies have found that tablet devices and digital pens can support learning (Purba and Hwang, 2018; Urban, 2017).

We conducted an extensive literature search and found very few articles describing the use of digital pen technology for chemistry lessons, and those we did find were mainly descriptive studies or anecdotal, and did not adopt rigorous research methods. Nevertheless, the authors of these articles indicated the benefits of using digital pen technology, particularly for chemistry learning (Morsch and Lewis, 2015; Urban, 2017). Urban (2017) presented an evaluation of Technology-Enhanced Learning (TEL) pedagogy which involved the integration of a pen-enabled devices into a number of chemistry courses. The TEL pedagogy, pen-enabled device and cloud-based teaching methodologies were adopted to improve chemistry students’ learning experiences. Through the TEL pedagogy and the digital pen devices, students were able to engage in learning in a number of ways. For instance, they could engage in visual annotations, real-time annotations, problem solving (by providing their own written response via the pen) and linking notes. The TEL pedagogy is especially critical for learning concepts in science such as organic chemistry which requires annotations of structures. Based on student surveys of six undergraduate courses that used TEL innovation, student experiences were enhanced and positive feedback was received from the students. However, current knowledge of how digital pens facilitate learning is still limited, and it appears that very few robust research methodologies have been applied.

It appears that in-depth analyses of student and teacher use of these digital technologies are relatively rare, particularly, observational data from naturalistic settings that capture the actual experience of learners and their tutors when using tablet devices and/or digital pens. Learning is complex and should be studied in context (Greeno, 2011). In this study, we sought to address the gap in current research through employing an “enhanced” observational approach which involves the collection of video footage of teaching and learning activities, supplemented with still photographs. These data were then interpreted through the lens of Activity Theory (Engeström, 1987, 2001).

### 2.2 Activity theory

Activity theory has been identified as a useful framework for research on mobile learning technologies (Chung et al., 2019). However, the efforts to use activity theory to analyse learning with students using tablet devices are still fragmented. Little is known about how it can effectively be used as a framework for emerging learning. It appears that there have been no studies that deploy activity theory for examining the use of tablet devices, in particular integrating digital pen technology into chemistry lessons. As we aim to unravel the dynamics of using tablet devices and digital pen technology in chemistry learning, we believe that activity theory as a framework of analysis is likely to provide a more systemic perspective of the phenomenon. The analytical value of activity theory is that it has the interpretive power to explain a particular phenomenon by considering the interactions of systems within the activity and the interrelationships among related entities.

Activity theory has been employed as a theoretical framework in many disciplines and contexts to capture the complexities involved in learning (Kirby and Anwar, 2020). Research studies using activity theory tend to emphasize issues such as how technology mediates knowledge construction, the complexities involved in technology-related learning, and informing the design of new learning environments (Rambe, 2012). Activity theory offers us a fruitful way to understand the interactions of key components of learning in a natural setting. Activity theory as a theoretical framework supported our study as it focuses on how students interact with tools to achieve outcomes and how the division of labor, rules and communities influence the activity system leading to outcomes. In this study, we used the activity theory to examine emerging learning with tablet devices and digital pen technology in a chemistry classroom so as to provide a comprehensive understanding of how such technology could foster learning. Our aim is not to report a surface understanding of how students learn, but to document the intricacies of learning that might take place.

Activity theory was first proposed by Vygotsky (1978) as he regarded human interaction with the social world as being mediated by semiotic tools and signs. Since then, activity theory has been modified or broadened by various researchers to optimize its usage in analyzing systems. In this study, we selected Engeström's (1987, 2001) model as it has been widely adopted to analyse how systems are influenced by the interactions among their various components (Holen et al., 2017). Activity theory is often used to evaluate an activity system by identifying tensions or contradictions among its different components that could cause the failure of the system (Holen et al., 2017).

**Figure 1:**
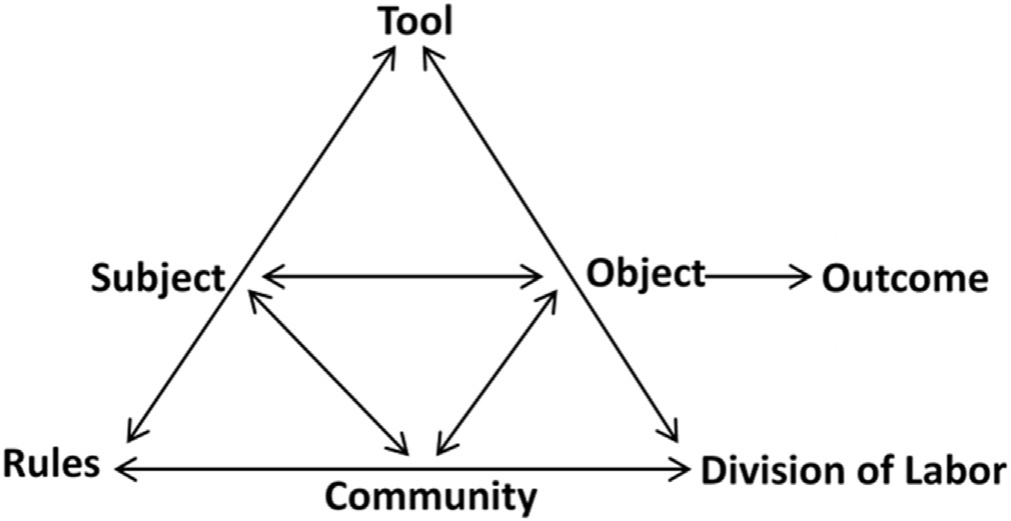
Model of activity theory.

**Figure 2:**
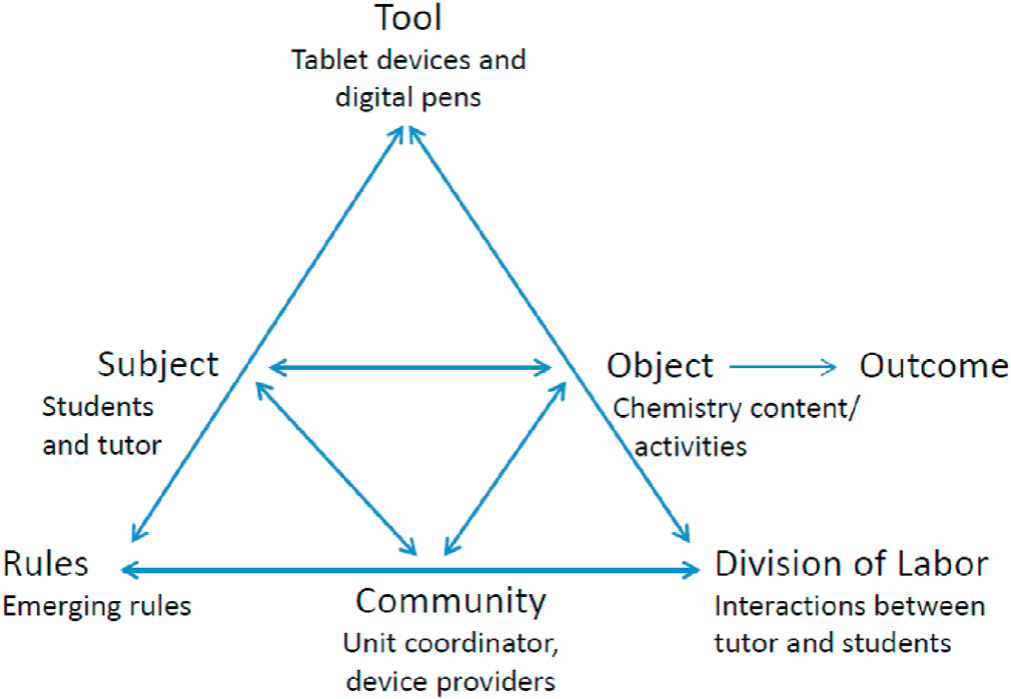
Components of the system in this study.

In the activity theory model explained by Engeström, there are six main components within a system and the activity is an outcome of the interactions of those components. In this context, chemistry lessons in Organic Chemistry are the activity system. The main components include subject, objects, tools, rules, community and division of labor (Figure 1). The subsequent figure (Figure 2), shows the components of the system in this context, as it is the framework of our analysis. Subject usually refers to an individual or a group of individuals who are engaged in the activity, while object is the aim or goal for the activity. Subjects are identified as the students and tutors. The object in this activity system included chemistry content and in-class activities. These were considered as representative objects as they were the most frequently used objects to transform all the learning events into learning outcomes. Tools are what the subjects used to achieve the goal; in this context the tools are the tablet devices and digital pen. Rules refer to the constraints that regulate the components and operations within the system, and lastly, community refers to the characteristics of the community where the activity occurs. In this case, one of the key rules refers to the manner in which the subjects operate the tablet devices and digital pens. The community refers to those who negotiate and mediate the rules that describe how it functions, and in this context it includes the providers of the tablet devices and the coordinator of the chemistry unit. Division of labor refers to the roles and relationships within the community that affect task division. In this context, the focus was on the interactions among the students, tutor and coordinator.

### 2.3 Interpretational power of video data

Multiple studies in the context of education have highlighted the interpretational power of video data (Alibali and Nathan, 2007; Lemke, 2000; Lehrer and Schauble, 2004), to assist researchers to acquire in-depth insights into phenomena being studied. For instance, Sakr et al. (2016) provided an example of video analysis that examined students' emotional engagement in history learning. In another similar study, Hollingsworth and Clarke (2017) reported that videos have been regarded as a catalyst for teachers’ professional learning (Hollingsworth and Clarke, 2017). Visual data are ideal for studying situational dynamics as compared to retrospective interviews and surveys which are not powerful enough to capture or record details about situation (Nassauer and Legewie, 2018).

The central tenant of this study is to closely examine students' uses and interactions with tablet devices and digital pens during classroom lessons, and to capture the situational dynamics of students' learning experiences, especially when they are tasked with studying complex and abstract concepts in organic chemistry. In this study, video recordings are a major source of data as they offer a form of close documentation and observation (Derry et al., 2010). Video-recordings of observations may capture the multiple elements in complex environments and in order to meaningfully analyse the data, we adopted the perspective from perceptual psychology in which video segments represent *events* (Zacks and Tversky, 2001), which have underlying structures reflecting multiple parts and timescales (Lemke, 2000). Moreover, because this study focuses predominantly on learning with tablet devices and digital pens, we have identified timescales that capture students’ use of technology for learning chemistry as “critical events” for analysis (Powell et al., 2003). Using the deductive approach (Derry et al., 2010), we focused on students and tutor interaction with the tablet device and digital pen, and we then strategically selected relevant video segments for analysis. This was followed by using “content logs” which consist of descriptions of the video segments for indexing purposes (Derry et al., 2010). Referring to the approach by Alibali and Nathan (2007), we then developed a coding system consisting of key elements such as finger/pen movements, student interaction with tutor/peers, and student response to feedback in order to categorize the observed activities and calculate the frequencies of occurrences. While analysing the video-recordings, we adopted some of the principles outlined by Nassauer and Legewie (2018) on using video data as a methodological framework. More specifically, we adopted the criteria for validity which are: optimal capture of duration, space, and participants, assuring the quality of recording and using triangulation.

## 3 Methods

### 3.1 Research design: exploratory case study design

This study adopted the general tenant of an exploratory case study. Given that the use of tablet devices with digital pens for learning is an emerging area of research, an exploratory case study design is appropriate. Because of the exploratory nature of this study, the questions asked in the interviews and the way we conducted the observations were meant to open up the possibility for further examination of the phenomenon observed.

### 3.2 Participants and setting

We used the purposeful sampling method which is commonly used in qualitative exploratory research for identifying and selecting information-rich cases for the use of limited resources (Patton, 2002). Through this method, we identified groups of students who had experience using mobile technology, as well as those who were first introduced to organic chemistry (Creswell and Plano Clark, 2011). This research has obtained ethics approval (H1 1929) from the Western Sydney University Human Research Ethics Committee.

Prior to our study, we communicated with potential participants and briefed them on the purposes and procedures of the study. Only students who were willing and available to participate were included in our data collection.

At the beginning of the 2017 autumn semester, we recruited undergraduate students enrolled in the Organic Chemistry unit in the School of Science and Health at a university based in Greater Western Sydney. Discussions took place between the researchers and unit coordinator to determine the feasibility of conducting research in one of the tutorial classes. After identifying a particular class that was starting their unit on essential chemistry II at the beginning of the semester, email invitations with a participation information sheet and consent forms were sent to the tutor and students. In all, 19 students volunteered to take part in our study. All participants were above the age of 18. The unit coordinator, tutor and students who volunteered were provided with shopping vouchers at the end of the study as an acknowledgement of their time and effort. During the classroom and lab observations, participating students were gently reminded to congregate on one side of the classroom or lab so that the researchers did not include non-participating students in their observations. Students in this unit were each equipped with a tablet device. As our research partner, Microsoft provided each participant with a digital pen and keyboard. During their chemistry lessons, the students accessed ChemSketch (free software that allows users to draw chemical structures) and Microsoft OneNote which were installed on their tablet devices.

### 3.3 Capturing learning and teaching in action

To capture a more realistic picture of Chemistry learning, four classroom observations were conducted, of which two were conducted during tutorials and the other two in the science laboratory. Each observation lasted approximately 45 min. At least two researchers were present at the research sites for each observation session. To capture the students and their tutor using the devices in real-time, two video cameras were set up during observations. As we sought to understand how the tutor uses the tablet devices and digital pens, a video camera was set up to capture her actions. The other video camera was mobile and moved around with the researcher, capturing participants in action and zooming in on particular students or groups of students when they were actively using the tablet devices. Still photographs were also taken for the purpose of comparison with the video data and observational field notes, and as a form of triangulation. Although photographs were not the main source of data, they were used for comparison with the video data for significant themes. In addition, as this study focused on the use of the tablet device with digital pens, it was paramount to capture the actions of the participants for an in-depth analysis of the process of learning. Both the video data and static images provided the researchers with a gateway through which to examine the bodily movements of the students and their tutor that corresponded with the tools and learning content.

Three researchers (authors A, B and D) completed detailed observation field notes independently and immediately after the observations for data analysis. An observation checklist was used during the observation to record instances when the student participants and their tutor used the tablet devices and digital pens for learning and teaching organic chemistry at macroscopic, symbolic and micro levels of learning. This checklist (Table 1 for part of the checklist used) was co-developed by the researchers and the unit coordinator based on the key concepts in organic chemistry. A list of indicators was used for this purpose. For instance, at the macroscopic level, an indicator would be “Using the tablet device/and with the pen to manipulate information to understand chemistry concepts “use the tablet device to do calculation and plot graphs),” and at the symbolic level, “Using the pen to represent symbolic structures of chemistry concepts/chemical reactions (draw molecules),” whereas for micro level understanding, an indicator would be “Using the pen to manipulate the sub-microscopic structures of chemistry concepts.”

**Table 1:** Observation checklist.

Indicators (Checked observed indicators)		Comments (Explain and support the observed indicators)
**MACROSCOPIC LEVEL**		
• Using surface device to understand chemistry concepts (quizzes).	( )	The tutor showed students how to read the chart through OneNote. She also showed how students can draw information from the chart for the calculation (deduce the structure of the compound)
• Using surface device to observe chemical reactions (video watching).	( )	
• Using the device/and with the pen to manipulate information to understand chemistry concepts (use the surface to do calculation and plot graphs).	( ✓)	
• Using the device to learn techniques (how to perform specific tasks, doing while watching the videos).	( )	
**SYMBOLIC LEVEL**		
• Using the device and or with pen to understand symbolic levels of chemistry concepts.	( )	Tutor repeated what she has shown in the previous lesson-drawing of molecules. In this lesson, she emphasized on the arrows that shows the reactions. She taught students how to draw arrows in Chemsketch.
• Using the surface device to interpret chemistry concepts (more of a symbolic and microscopic – watching videos and programs to illustrate what is happening to atoms and molecules).	( )	
• Using the pen to represent symbolic structures of chemistry concepts/chemical reactions (draw molecules).	( ✓)	
• Using the pen to manipulate the symbolic structures of chemistry concepts (drawing the molecules, mechanisms).	( )	
**MICRO LEVEL (SUB-MIRCOSCOPIC)**		
• Using the device and or with pen to understand the micro level of chemistry concepts	( )	
• Using the pen to manipulate the SUB-MIRCOSCOPIC structures of chemistry concepts	( )	
• Using the surface device to interpret chemistry concepts (more of a symbolic and microscopic)	( )	

In total, 44 video clips were captured, constituting approximately 5 h of video recordings (excluding activities not related to learning, such as arranging the apparatus in the lab), and 150 still photos were taken.

### 3.4 Interviewing learners and the instructor

We conducted semi-structured interviews as this method ensures that the conversations between the researchers and participants adheres to the research objectives and aims (Becker et al., 2012), and at the same time, it offers participants an opportunity to flexibly expound or discuss any issues. Such data collection method also provides useful information to compliment observations (Creswell, 2012). Towards the end of the study, semi-structured interviews were conducted involving eleven students, one tutor and a unit coordinator. In total, three focus groups with students, one phone interview with a student (who was absent from the focus group interview), one interview with the tutor via video conferencing, and a face-to-face interview with the unit coordinator took place. Each focus group session or interview lasted between 30 and 50 min, and all were audio-recorded. The purpose of the focus groups and interviews was to explore the experiences of the participants; hence the interviews focused on asking participants to describe their experiences with the aid of probing questions such as “How do you use the devices to draw graphical representations of the chemistry concepts?” Because the interviews were carried out at the end of the study, the researchers were able to record classroom interactions, and used these as prompts to help the interviewees recall and reflect upon their own learning. For instance, one of the questions asked was, “Previously you used your finger to draw and write the equations; how did you find using the pen to draw and write in comparison to using your finger?”

### 3.5 Analyzing data

**Figure 3:**
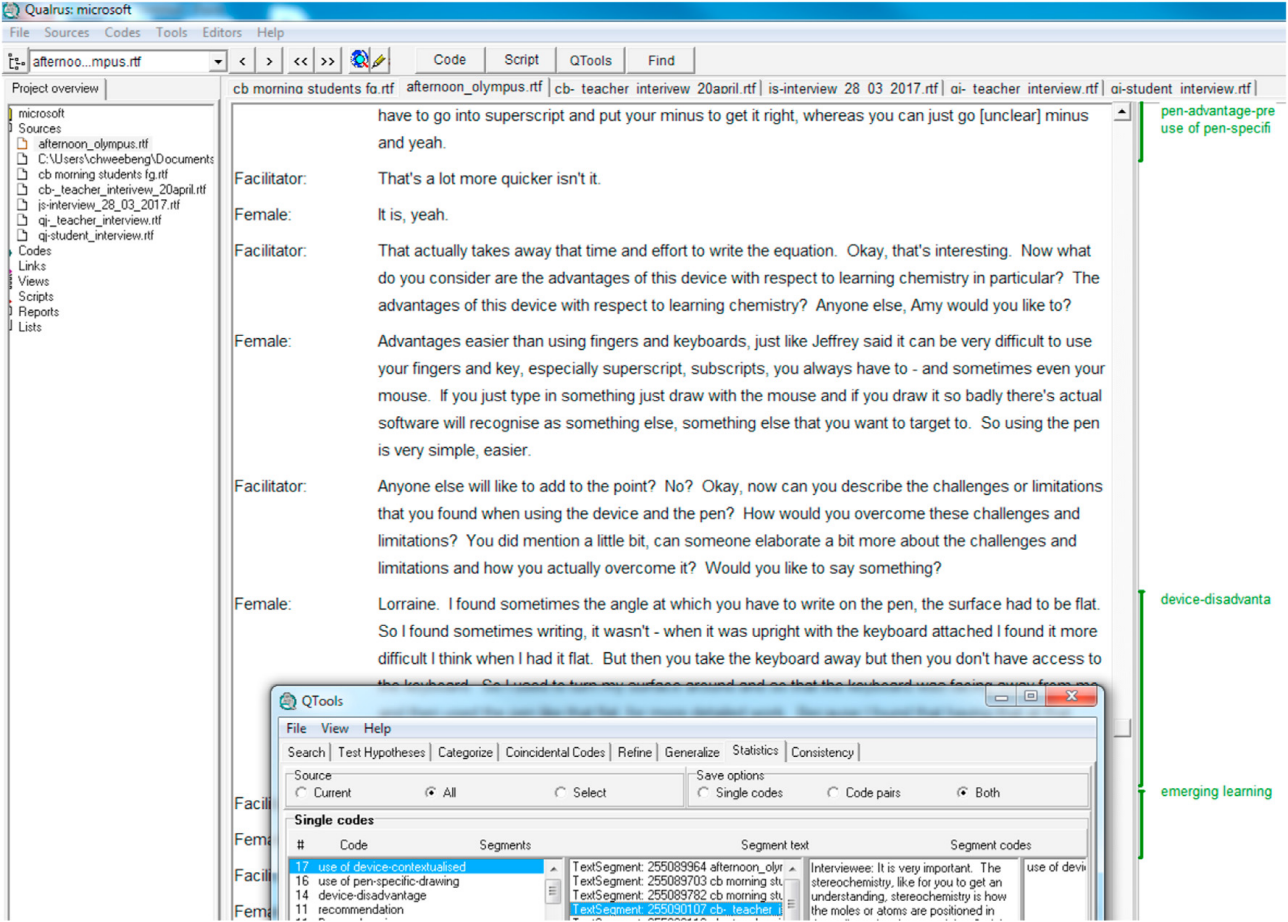
An example of using Qualrus for data analysis.

The interview transcripts and observation field notes were analysed concurrently using the software, *Qualrus*. Figure 3 shows an example of how we used the software for data analysis. In this project, we adopted some grounded theory principles. The analysis followed the guidelines suggested by Strauss and Corbin (1990), and we mainly used open and axial coding. Open coding involves the breaking down, comparing and categorizing of data. For this coding process, specifying the characteristics of categories is crucial. Initially, general terms such as “advantage of pen” and “use of tablet device” were used to describe segments of data. Sub-categories emerged from the general categories. In axial coding, the researchers re-gathered the data and put them back together in new ways by making connections between a category and its subcategories. The video data and still photos were used in the process of coding for triangulation purposes. In our case, as we were interested in understanding the phenomenon through the lens of activity theory, we did not resort to using selective coding as suggested by Strauss and Corbin (1990) for allowing a core category to emerge. After the axial coding, we used activity theory as an interpretive lens to explain the phenomenon being examined. Specifically, we examined the existence of interactions between the components. For instance, as “use of tablet device” was connected to the sub-categories “tool for reflection” and “reduces cognitive load”, we then established the interaction between “subject” (student), “tool” (digital pen) and “object” (reduces students’ cognitive load) by building the relevant theme “fostering focused learning” to explain such interaction.

## 4 Results

### 4.1 Fostering focused learning

Through the interaction between tool, subject and object, one of the key themes that emerged was focused learning. As the use of the device (with the digital pen) is contextualized, students were focused in their learning. This was evident when one student commented that through using the digital pen, he was able to learn the difficult process of constructing the molecules as he was able to concentrate and he used an interesting analogy to describe this process: “it's like using a recipe for cooking rather than going to a restaurant and sitting down to just eat.” Another student provided a lengthy explanation on how the digital pen helped him to focus through manipulating his written notes to highlight the important information and as a result, he could invest his energy and time to understand the content whereas he “can't do the same with a pen and paper.”

Our findings suggest that tablet devices and digital pens reduced students’ cognitive load so that they are able to stay focused, have a clearer understanding of the concepts, interact with their tutor, and engage in **reflective thinking** which leads to better understanding. While one student commented in the interview that the digital pen technology helped him to reflect and think about what he had understood, another student commented:

“*So if the teacher was explaining say the stereoisomers…She will actually quickly just scribble on the board just to show us how this reaction takes place…because I'm a very visual person I love to draw the actual structures and how they are going from one form to the other and then becoming a final product. So I find it really easy to draw it in the Surface device.*”

Interestingly, we found that a number of students tended to use the digital pen as a tool to engage in reflective thinking. This happened when they were not writing nor drawing but using their digital pens pointing and tapping at text on the Surface and pausing in between these actions. One student mentioned that because the digital pen is easy to use, she can “write freely and pause and think when listening to the tutor.”

The technology necessarily enables students to learn through visual simulation which is important, especially when laboratory work is required. For instance, one student commented that:

“*You can't learn chemistry in the classroom. You have to go to the lab….because if you just try to recall something that doesn't make sense, you'll never remember it. But when you do it, you understand what's going on. So I think the pen and the device are breaking the linkage between what's written on the paper or what's taught in the classroom and what's happening in the lab. You can see the laboratory apparatus on ChemSketch. You can go to the database on the software itself and have a look and sense and feel how the apparatus works in a real lab situation.*”

Although students can also use Chemsketch on their computer or laptop, the tablet device with the digital pen enabled them to interact more efficiently with the technology through effortless manipulation of the variables.

Another student also commented on the **flexibility** of the technology that enabled her to learn more effectively:

“*If you have that in your notes, let's say in your Surface tablet, whenever you come to it, rather than staring at a blank picture or a static picture, you can see the animations and that helps you record information a lot quicker.*”

Similarly, another student echoed this view by commenting that as she watched how the tutor sketched the molecular structure, she was able to add to the drawing using her own device. Another student indicated that when he used the digital pen for note taking, he found that he could retain information more easily as compared to typing notes. During one of the observations, we noted that a student used the digital pen for writing down notes and annotating key concepts while the tutor was teaching.

One of the key issues that we looked out for during observations was how focused the students were when performing their tasks, as being focused can be an important factor in student achievement (Usart et al., 2013). Focus is interpreted as students’ ability to concentrate exclusively on performing the necessary learning activities (Cardoso-Leite and Bavelier, 2014). While on-task behavior (such as digital note-taking in this instance) is time spent on learning activities, off-task behavior (such as talking with peers or playing with cellphones in this instance) is time spent on activities other than the learning tasks (Karweit and Slavin, 1982). Based on our analysis, it is evident that the tool, which is the tablet device, coupled with the digital pen enabled the students to regulate their behavior as they stayed focused and task oriented throughout the learning process. In fact, this finding is congruent with what we found in our video analysis. Throughout the four lesson observations, we have no record of observed off-task behaviors.

### 4.2 Use of the device is contextualized

Again, when we examined the interactions between subject, tool and object, we concluded that the use of the technology for learning is contextualized. There were 16 instances of coding that revealed that the students were consistently using the digital pen for specific types of drawing and for **precision** (Figure 1). An extract from one of the participants illustrates this:

“*Each molecule in organic chemistry has a charge. What causes the action is a charge. So that molecule gives away its charge to a molecule depending on certain rules and it follows steps. [It's] well defined, well known or predefined; well-known steps are called a mechanism. With the pen, it's much easier to learn the mechanism because there are intermediates - during the emergence of the intermediates, another action is going to happen and that new action is based on a new mechanism…you need to know what is potassium permanganates, what's going to cause the oxidation reaction. You need to know the steps of the mechanism, how it's going to happen, what's going to happen in the end, and how the new molecule is going to appear. So I find the pen and the device is very helpful in learning mechanisms.”* Similarly, another student commented that: “*I draw the chemical reactions so that it makes more sense and how there was less friction, so I find it easier drawing it, like faster.*”

During our observations, we noticed that when drawing molecules using the Chemistry software, ChemSketch, most students preferred to use the digital pen as it was much easier to draw molecules than using their fingers.

In chemistry, precision in drawing of molecular structures is critical to learning. The functionality of the tablet device together with the digital pen afforded precise drawing. This is reflected in a comment made during the interview:

“*The more important thing about the pen is in organic chemistry. When you twist the mechanism, sometimes the arrowhead, the tip and the head - like the tail and head of the arrow… when you do it by hand, you wouldn't be able to figure out whether it's coming from the carbon and going to the oxygen…But when you do it with something neat like that (using the Surface and digital pen), it's much neater so you can work it out.*”

The unit coordinator shared a similar view when he mentioned that the arrows of the molecular bonds are very important as they show the movement of the structure.

Our finding is consistent with other studies given that other researchers have also highlighted that the two key features of mobile learning are “action” and “contextualization” (Chung et al., 2019). While “action” refers to the anytime anywhere teaching and learning, “contextualization” refers to the seamless integration of real-world learning and digital content.

### 4.3 Use of the tablet device encourages emerging learning

Throughout our observations and our analysis of the intricate interactions among tool, subject, division of labor and rules, we noted that the students devised new ways of approaching learning with the tablet device and the pen. In other words, the technology that the students were using stimulated them to construct their own learning content and process (Enonbun, 2010). One student mentioned that she was able to **collaborate** with three of her peers in a Microbiology class on a group task after she learned how to use OneNote. Transferring her skills to another learning context helped her to work effectively on group tasks as she said:

“*So four of us sat around a table; it was on everyone's device, and then everybody could see the one document and then it was shared easily and instantly*.”

We observed that impromptu collaborative learning occurred across all lessons when students took the initiative to help each other solve problems on the tablet device. **Adaptive learning** also emerged from the learning process. It was interesting to note that to overcome some of the challenges of using the tablet device, students devised their own ways of coping. For instance, one student mentioned that:

*“I found sometimes the angle at which you have to write with the pen, the surface had to be flat. So when it was upright with the keyboard attached I found it more difficult I think than when I had it flat. But then you take the keyboard away but then you don't have access to the keyboard. So I used to turn my Surface around so that the keyboard was facing away from me and then used the pen like that flat, for more detailed work. Because I found that having that at that angle the pen just wouldn't work sometimes. So…I turned it around so obviously the screen turned so that was okay*.”

In another instance, we observed that one student juxtaposed two documents on his tablet device. With one ClassNote PDF which the tutor was working on open on the left of the student's screen, and a ChemSketch document open on the right side of the screen, the student was using the digital pen to draw the structure of the molecules based on the tutor's explanation.

Although the community in this context referred to students, tutor, unit coordinator and the service provider, the interaction between the unit coordinator and service provider was not explicitly captured due to the nature and timeframe of the study. However, we have documented instances where the tutor was able to assess her students’ level of understanding through the device. When interviewed, she mentioned that to help students understand more clearly the key concepts, she could use the tablet device and project her feedback for the whole class to discuss. Perhaps this becomes a way to **communicate** and build better rapport with students. While not standing in front of the class, the tutor would walk around to assist students with their tasks. There were constant interactions between the tutor and her students, and most of the time, the device was used as a platform for students and tutor to co-build knowledge. We observed that, in many instances, the tutor and her students were using their digital pens and tablets to write and draw while interacting and discussing.

With the flexibility that the device offers, students’ learning is enhanced through **multiple representations** of the learned concepts. They were able to manipulate the learning space for effective learning. For instance, one student mentioned that:

“*It's interesting because you can even have sort of input videos, even GIFs or [JIFs] they're called, into let's say your notes. That way, you sort of have an animated version of a chemical reaction and it shows how electrons move to which place and what reacts with what. I would say it would provide a good summary as well. This is opposed to a piece of paper when you're just looking at the image. Especially when you're doing revision and you're like what's this image about? Whereas if you see a GIF, you can see everything* …” In this example, the student used a multimodal approach for visualization, leading to effective learning.

### 4.4 Use of device is dependent on individual differences

The interaction between tool and subject brought up the importance of rules. According to the embodied perspective (Macedonia, 2019) letting students use gestures to simulate the movements and allowing them to manipulate the virtual objects through physical actions may promote student learning. As Chemistry learning focuses much on symbols and diagrammatic forms of expression, handwritten elements of technology enable problem representation and interpretation of meanings.

Our findings revealed that students’ use of the tablet device is dependent on individual differences such as **prior knowledge** of the device and learning preferences. We noted that a number of students in this project were more accustomed to learning through the visual medium. For instance, one student mentioned that:

“*I am a visual learner. So sometimes instead of just writing words, I use just pictures to identify what's happening. So instead of saying, for example, this to there, I just do an arrow and maybe whatever - if it's a molecule, then I would be drawing the two molecules and just an arrow just saying - to pretty much save time*.”

There were students who preferred capturing their notes digitally and in this case, the device improves their efficiency in learning as one said:

“*I don't type fast…so, I generally write. I rarely use devices to actually take my notes. But with a device that comes with a digital pen, I can actually write it down digitally and because I do wish to write my notes digitally…I go back home I write my notes digitally by typing it, but it takes more time. To have a device that I could use to write with the pen saves me time when I get back home to write notes*.”

During lesson observations, we noticed that students who had been using the tablet device for some time, continued to use it for this project and were efficient in manipulating the technology. For instance, several students who were more advanced in using the tablet device were able to operate it device without any technical issues. We also recorded instances where female students tended to use colors in their drawings though there were no such records for male students.

Our video data also revealed that a number of students consistently used their fingers, the keyboard and the digital pen at the same time. They seemed to do so following an observed particular pattern. Specifically, students tended to use their finger or digital pen to scroll, but when it came to enlarging images, they switched to using their finger, but switched to use the pen to tab on key features on the tablet device or to point at a particular object. For quick typing they tended to use the keyboard, while when it came to writing and pondering at the same time, they tended to use the digital pen. Interestingly, one of the interviewees commented that “typing is more mechanical than using the pen and paper”; perhaps this is one subtle rule that was commonly used by the participants.

### 4.5 Challenges in using the device

No activity system is free from limitations. Despite the positive responses we gathered from this study with regard to the use of the device for chemistry learning, we also identified tensions among the participants. Such tensions arose mainly from two aspects. First, the current limitations of both the tablet device and digital pen. These included the slow response time of the digital pen, its limitation in terms of scrolling, unstable connection and delayed time in saving. Among these, one of the more intriguing limitations is the limited synchronicity between the current design of the tablet device and natural human movement of the palm and fingers. One participant described her experience of using the tablet device: “like just writing, maybe so that it does not react when you have your palm touching the screen…because that's generally how people write. When they write, they generally have part of the palm touching the surface. But since this is a touch screen in the first place, so when you try to do that, it automatically reacts.” Because cognition is grounded in action, as argued by de Koning and Tabbers (2011), the more we need to consider the design of the learning devices from a more systemic perspective. To elaborate, devices that are designed for learning must also be tested for natural movements and actions which synchronize with thought processes.

The other aspect of tension relates to end users’ expectations of future devices. Participants were keen to use the tablet device and digital pen for learning and were also quick to suggest ways to improve the mobile technologies based on their expectations and visualization of how they should be designed. For instance, participants argued for more customized features for the digital pen including options for left-handed users and appropriately designed sensors for more seamless actions. One participant commented that the digital pen is currently so sensitive that it was still working (e.g., opening up tasks on the screen) when it was not intended to. For this reason, participants suggested that the digital pen be able to have a “sensitivity setting that you can actually set to a low or high sensitivity for the pen.”

## 5 Discussion

Through an exploratory case study approach and with the use of Activity theory as an analysis framework, we sought to answer research question 1 which was to understand how the tablet device coupled with digital pen technology facilitates university students’ learning of chemistry. Using activity theory as the interpretive lens in this context provided a systemic perspective on the phenomenon being studied. Given that there are few studies that have described the use of digital pen technology for chemistry learning, and the lack of research rigor in these studies, our study may provide important insights into the complex nature of learning abstract chemistry concepts with digital pen and tablet technology. Through examining the interactions between the key components of a system, we were able to develop a deep understanding of how such technology can facilitate chemistry learning. Several key themes emerged, highlighting the interactions of the key actors within the activity system, while tensions were also identified. These findings were unlikely to be possible if we had not used the activity theory to understand the complex nature of learning. For instance, we were able to conclude that the students not only used the digital pen and tablet device for drawing molecular structures and for note taking, but they were also using it as a tool to engage in reflective thinking. Such interaction between subject and object may potentially subtly alter the learning outcome, and educators need to be aware of this dynamic process in order to respond to the changing nature of classroom learning. Studies on technology for learning commonly report the effective use of particular tools or groups of tools for learning. However, in our study, we reported that students devised their own ways of coping to overcome the challenges of using the device. We recorded a number of instances where adaptive learning emerged from the learning process. For instance, some students described extensively on how they manipulated the position of their devices or the way they present documents on their devices to suit their learning needs and preferences. Again, such observations reveal the interaction between subject and object and how rules are being altered in this interaction process. When rules are modified during the process of learning, the pedagogy of teaching may have to be adjusted to respond to the needs of the students. On a broader perspective, higher education institutions may need to evaluate their current instructional practice to determine whether there are opportunities for flexibly allowing the interactions among the main components of a system to emerge and subsequently modifying the curriculum accordingly. A challenge that lies ahead is to better understand how the digital pens and tablet devices shape the way educators perceive and design their teaching in other science domains, particularly areas that require the learning and manipulation of abstract concepts. Educators may need to re-think the application of mobile technologies such as the digital pen and tablet technology as they no longer limit themselves to support knowledge retention or creation, but rather facilitating and propelling emerging learning based on the dynamic interactions of the key components of a learning system. The continued exploration on the use of the digital pen and tablet technology may enable educators to redefine their pedagogical practices and prompt institutions to consider implementing policies around effective use of technologies for meaningful learning. Particularly, the requirements for the generic university classrooms may need to be modified for the integration of the digital pen and tablet technology into the everyday classroom learning.

Our study has also highlighted the criticality of robust research design and analysis if the purpose of a particular study is to fully understand the affordances of digital pens and tablet devices in chemistry classes. Previous related reports have highlighted the benefits of such technology such as visual annotations. However, although we believe that these findings are important, the complexity of learning with the technology has not yet been fully explored. If we continue to conduct technology-related research from the lens of viewing technology as tools of learning, then we may not unearth the mechanisms of learning with technology. In our study, through activity theory, we were able to observe the interactions of the key components in the chemistry classroom and thus develop a systemic picture of learning with technology.

Learning is complex and most of the time contextualised. In our study, we documented the process of contextualised learning by students using tablet devices coupled with digital pens. This is especially true when learning requires the manipulation of technology for understanding abstract concepts. Our participants showed that when learning Chemistry, the combined use of the tablet device and digital pen fostered their learning, especially when precision of drawing of molecular structure is critical to learning. We have documented instances of students' preference in using the digital pen for precise drawing than their fingers. Such instances highlight the key features of mobile learning-contextualization and action. This observation concurs with the notion of “affordances” of technology. The advancement of technology does not necessarily change the theories of learning, but rather reinforces it. Although it was not the intention of this study to examine students' learning outcomes, we were confident that the combined usage of the tablet device and digital pen does add value to students' learning. Not only were we able to hear from participants and their tutor about the advantages of the technology for learning, we consistently observed that the participants were highly focused during the learning process. Our video analysis also revealed that there were no off-task behaviors throughout all lesson observations. With their learning contextualised, the students were focused in their learning. The technology effectively reduces students’ cognitive load so that they are able to stay focused throughout the lessons. In essence, we found that the tablet device together with the digital pen enabled the students to focus on their learning, and they were able to learn through visualisation and manipulation of the technology. This finding is aligned with Oviatt (2013) position on the importance of using interfaces that support expression of non-linguistic representations. This is especially apparent when learning is contextualised and in the context of learning abstract concepts like those in chemistry which require multiple representations for better comprehension. Educators may consider tapping into the affordances of the tablet technology for the learning of abstract or difficult concepts, especially in helping students to manipulate and interact with variables and constructing visual representations of their understanding.

Our second research question aimed to explore students’ overall experiences of the use of digital pen technology in their learning. Related studies (Chung et al., 2019; Crompton and Burke, 2018). have consistently found that the use of technology enhances experience, motivates or engages learners. Such findings usually portray a rather “linear” relationship between technology and learners. However, in our study, we documented the process of “emerging learning.” In other words, students were not passive recipients of the technology. There was evidence that they devised new ways to approach learning with the device and the digital pen. The technology that the students were using stimulated them to construct their own learning content and process (Enonbun, 2010). They were able to manipulate the learning space for effective learning. For instance, we observed that impromptu collaborative learning occurred across all lessons as students took the initiative to engage in collaborative problem solving. This is an important finding as it suggests that learning is a dynamic process and that learners constantly adapt to the process through adjusting their learning behaviors. This might mean that technology must allow for the evolution of emerging learning.

Most recent literature on digital pens (Huang et al., 2017) has suggested that mobile technology can promote learning attention and motivation and, when combined with collaborative problem solving, it might improve learning outcomes. Adding to these known findings, our research highlights the importance of taking into account individual differences given that learning is often a complex process.

## 6 Conclusions

The research presented here contributes to a growing body of knowledge on the nuances and affordances of mobile technologies in educational settings. The application of activity in this research provides systems-based insights into interplay of various factors that underlie students’ learning experiences with mobile technologies. Before discussing directions for future research it is important acknowledge limitations of the research.

## 7 Limitations

One of the challenges we faced when conducting this research was recruitment. Some students were reluctant to participate in this research as they were hesitant to be observed and interviewed. Despite this challenge, we were able to proceed with the study, and eventually collected ample data for analysis. It would be appropriate to design this study as a quasi-experimental study so that we could better understand the influence and impact that the digital pen and tablet technology has on chemistry learning. However, such a design was not possible given the constraints we faced in terms of timetabling and recruitment. Our initial research plan included a full semester of data collection so that we might track the changes in students’ learning with the digital pen and tablet technology. Considering the complex nature of learning chemistry concepts, we decided to narrow our focus on a specific chemistry topic to gain an in-depth understanding of the dynamics of learning.

We chose to extensively use video recordings in our research in order to capture students' and the tutor's activities and interactions. We also aimed to construct a full picture of chemistry learning with digital pens and tablet devices through a combination of video recordings and still pictures. Although such data collection and analysis processes were laborious, it was necessary and meaningful, and it aligns with using activity theory as a theoretical frame.

### 7.1 Future directions

As this is an exploratory case study and was carried out in the context of chemistry learning, it might be beneficial to conduct similar studies in other subject disciplines such as mathematics learning, business studies, engineering, etc. When we can understand more clearly the dynamic processes of learning that take place in different contexts, we can then modify the technology for meaningful learning to take place.

A quantitative study, with a larger sample of randomly selected participants using sophisticated statistical analysis such as structural equation modeling would also be beneficial for developing further insights into intricacies of learner uses of tablet devices and digital pens. Such a technique could possibly provide a statistically tested model for explaining the relationships between the tablet devices, digital pens, students’ experience, and their achievement scores.

## Declarations

### Author contribution statement

C. B. Lee, J. Hanham: Conceived and designed the experiments; Performed the experiments; Analyzed and interpreted the data; Contributed reagents, materials, analysis tools or data; Wrote the paper.

K. Kannangara, J. Qi: Performed the experiments; Contributed reagents, materials, analysis tools or data.

### Funding statement

This research did not receive any specific grant from funding agencies in the public, commercial, or not-for-profit sectors.

### Data availability statement

Data will be made available on request.

### Declaration of interests statement

The authors declare no conflict of interest.

### Additional information

No additional information is available for this paper.
